# Supplementary material for: Separating CO2 emission from removal targets comes with limited cost impacts
Source: Nat Commun. 2025 Jun 12;16:5298. doi: 10.1038/s41467-025-60606-7 (PMC12162817; doi:10.1038/s41467-025-60606-7)
Supplement: Supplementary file 1 — Supplementary Information [file 41467_2025_60606_MOESM1_ESM.pdf]

Supplementary Information for:

Separating CO_2 emission from removal targets comes with limited cost impacts

Anne Merfort [* 1,2], **Jessica Strefler**[1], **Gabriel Abrahão**[1], **Nico Bauer**[1], **Tabea Dorndorf**[1,3], **Elmar Kriegler**[1,4], **Gunnar Luderer**[1,2], **Leon Merfort**[1,2], **Ottmar Edenhofer**[1,5]

[*] Corresponding author: anne.merfort@pik-potsdam.de
[1] Potsdam Institute for Climate Impact Research (PIK), Member of the Leibniz Association, 14473 Potsdam, Germany
[2] Global Energy Systems Analysis, Technische Universität Berlin, 10623 Berlin, Germany
[3] Geographical Institute, Humboldt Universität zu Berlin, 12489 Berlin, Germany
[4] Faculty of Economics and Social Sciences, University of Potsdam, 14483 Potsdam, Germany
[5] Climate Economics and Public Policy, Technische Universität Berlin, 10623 Berlin, Germany

Supplementary item list

Supplementary Figure 1 2
Supplementary Figure 2 3
Supplementary Figure 3 3
Supplementary Note 1 4
Supplementary Methods 4
Supplementary Table 1 5
Supplementary Table 2 6
Supplementary Table 3 6
Supplementary Table 4 7
Supplementary References 7

Supplementary Figure 1

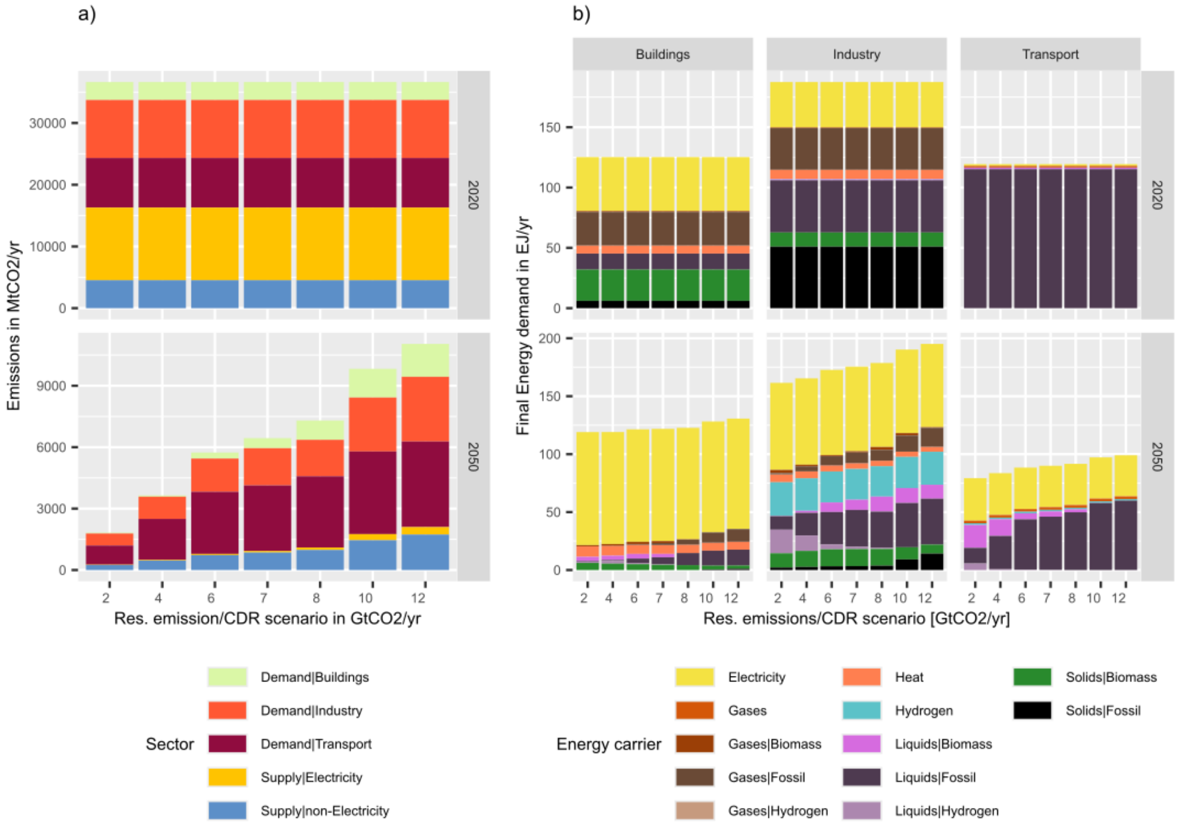

Supplementary-Figure 1: Sectoral distribution of residual emissions (panel a) and final energy demand (panel b). *Emissions are drastically reduced in all sectors and all scenarios, with the biggest share consistently remaining in transport due to the high remaining demand in liquid fuels for aviation and shipping. In all scenarios, the power sector is almost fully decarbonised and the demand sectors' transformations heavily rely on electrification to a similar degree across scenarios, suggesting that the potential is almost fully tapped in all scenarios. To bring down residual emissions, fossil liquids are increasingly replaced by a mixture of biofuels and synthetic fuels, with higher shares of synthetic fuels in scenarios with higher emission reduction (i.e. lower allowed residual emissions). Note that in REMINDv3.2.0[1], the distribution of different types of liquids across sectors is arbitrary and no conclusions can be drawn from a certain type of liquid being used in one sector rather than in another. The industry sector furthermore relies on hydrogen with similar shares on final energy across scenarios. To a certain extent, the energy system also relies on demand reductions in those sectors that rely on liquid fuel and feedstocks (transport and industry). This is cheaper than an even faster or earlier scale-up of synfuels. Source data are provided as a Source Data file.*

Supplementary Figure 2

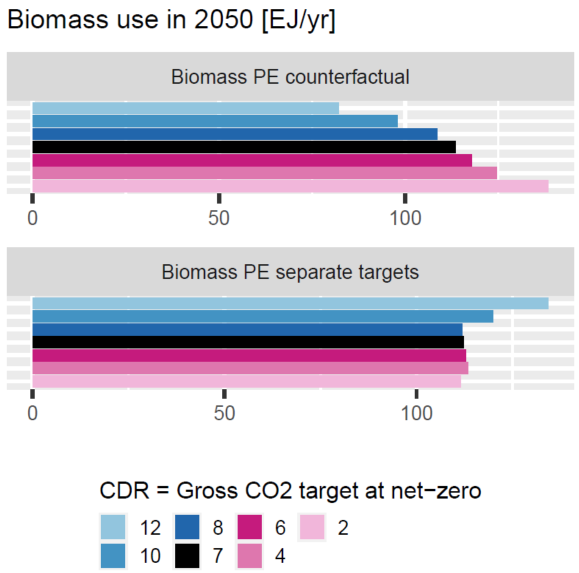

Supplementary-Figure 2: Biomass use in sensitivity scenarios with no constraint on biomass. *If the biomass limit of 100 EJ/yr is lifted, we observe almost identical biomass use of around 106 EJ/yr for scenarios with CDR targets from 2-8 GtCDR/yr. Significant increases only occur for the scenarios with highest CDR targets with 10 and 12 GtCDR/yr with 113 and 127 EJ/yr respectively. However, like the scenarios shown in the main analysis, different net-zero formulations lead to varying cumulative emissions until 2050. When contrasting the scenarios with separate targets with counterfactuals reaching the same cumulative emissions but with only a uniform carbon price, scenarios with equally tight CO2 budgets (pink scenarios) require more biomass while scenarios with comparably loose budgets require significantly less biomass (blue scenarios). Hence, the CDR target has an impact on overall biomass use but is by far not the sole driver of exacerbated biomass demand. Source data are provided as a Source Data file.*

Supplementary Figure 3

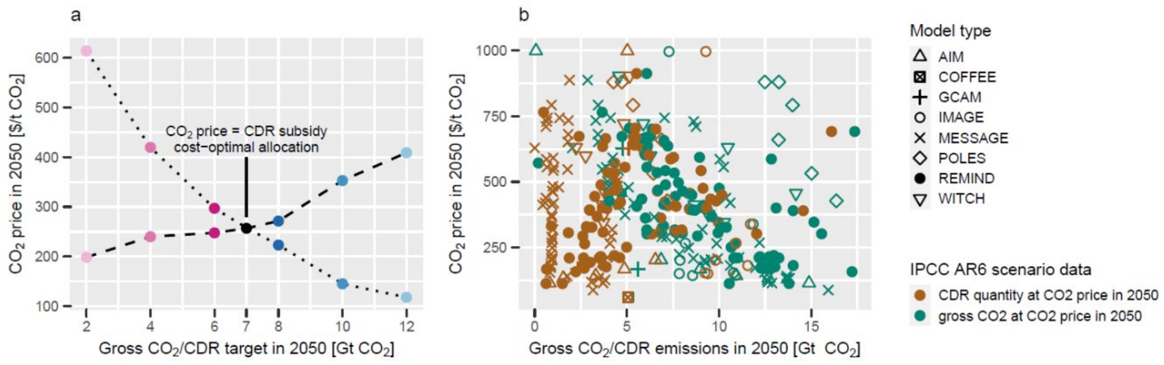

Supplementary-Figure 3: The prices derived from REMIND scenarios a) and uncertainties about future abatement and removal costs b). *To illustrate the inherent uncertainty of future carbon prices, we show the quantities of residual emissions (brown) and permanent CDR (green) at different carbon prices in 2050 from 165 IAM scenarios submitted to the IPCC 6th assessment report (panel b). Scenarios where filtered to only include all C1 and C2 scenarios, with carbon prices ranging from 50-1,000 $/tCO2 in 2050. Source data are provided as a Source Data file.*

Supplementary Note 1

Scenario rationale

As the future market efficient CDR contribution to net-zero is hard to predict with only current knowledge, there is a high chance that in case policymakers were to choose separate targets for emissions and removals now, they could over- or underestimate the CDR contribution. While in our modelling framework, 7 GtCDR/yr is the market efficient contribution, in reality this could very well be much lower. As we want to explore both, the consequences of higher and lower than market efficient contributions, we span the scenario range from 2-12 GtCO2/yr to still cover plausible ranges of relative deviations. Note that while the 12 GtCO2/yr scenario seems excessive in its reliance on CDR we still find it plausible that policymakers may overestimate optimal future CDR contributions by a factor of 2 (e.g. in a world, where the market efficient contribution of CDR to net-zero is really small such as 1 GtCO2/yr).

Supplementary Methods

Deviations from REMIND default assumptions

We make the following deviations from standard scenario settings in this analysis:

- We use a model version based on REMINDv3.2.0 that additionally includes a separate carbon market for novel CDR. The code is available on Github at https://github.com/amerfort/remind/tree/SepMark_REMIND3.2.0
- We exclude novel CDR from the tax on GHG emissions and add a complementary subsidy.
- Instead of a peak budget target, we prescribe explicit residual emissions and removal targets for 2050.
- The tax on net negative emissions is turned off to avoid additional modifications of the effective carbon price.
- Enhanced weathering is an available CDR option.
- Maximum annual primary energy biomass is limited to 100 EJ/yr for sustainability reasons.

Technoeconomic data on CDR technologies

In REMINDv3.2.0[2] there are 4 BECCS conversion routes that capture CO_2 released during combustion or the refinery processes that convert lignocellulosic biomass feedstock into secondary energy carriers: bioliquids, biogas, electricity and H_2. Which specific technology is economically most useful in a given scenario is not only dependent on the contribution to CDR but also on how valuable the secondary energy carrier is to the abatement side. Techno-economic parameters for bio-based supply side technologies with carbon capture are displayed in Supplementary Table 1.
In the model we represent a low-temperature Direct Air Capture technology following the specifications provided by Climeworks[3]. For DAC, learning is endogenous, with investment costs declining with increased cumulative capacity. For techno-economic parameters of DAC, please refer to Supplementary Table 2.

In REMINDv3.2.0 CO_2 transport and storage is represented as a single technology with techno-economic parameters provided in Supplementary Table 3. Geologic storage is available for all captured CO_2 (including fossil emissions and industrial process emissions). It only contributes to CDR if the origin of the captured CO_2 is atmospheric or biogenic. Furthermore, captured CO_2 from all source might not be stored but utilised for synthetic fuel production with subsequent rerelease to the atmosphere. Hence not all captured atmospheric or biogenic CO_2 contributes to CDR. The description of the additional tax on CCS is taken from Strefler et al. 2021[4]: "To reflect the risk of leakage and the associated possible costs, costs of improved safety criteria related to monitoring, reporting, and verification, and difficulties due to public acceptance, which are all likely to increase with deployment, the best estimate of costs for geological carbon storage is increased linearly such that costs are about 100% or 30 \$ $tCO_2{-}1$ higher at maximum deployment."
For still immature technologies, among others are all BECCS technologies, DAC, and CCS, there are mark-up costs for the near-term periods: investment costs are multiplied with 1.5 in 2025 and 1.2 in 2030.

Technoeconomic data on EW

The following description is taken from Strefler et al 2018[5] "EW is based on the acceleration of the natural weathering of silicate rocks, which is an integral part of the carbon cycle. In REMIND, we assume those rocks to be basalt, which is rich in phosphorus and potassium and contains very low concentrations of trace elements. 1 t of basalt binds 0.3 tCO2 in the weathering process. The basalt has to be mined, ground to small grain sizes, and spread on agricultural fields. We assume a maximum of 15 kg ground stone per square meter at any given point in time. Only the amount of basalt that weathers in a given year can then be replenished. The regional potential

for carbon removal depends on the agricultural land and the climate zone as this process is faster in warm and humid regions and amounts to a maximum of 4.9 Gt CO2/yr removed[6] (see Supplementary Table 4). However, the potential of EW is still highly uncertain. Weathering rates are so far only calculated and would have to be measured in field experiments. Also, the amount of stone that can be spread on fields is a rather conservative assumption and could be higher initially. In the long run, i.e. if applied over decades, it is unclear to what extent EW could be sustained without significantly changing the soil structure. Applying EW not only on croplands as assumed here but also on woods would increase the potential, but also the costs as spreading would become more difficult. Both uncertainties could change the total amount of EW, but would not have much impact on regional distributions as warm and humid regions would remain favourable. Economic costs are at about 200$/tCO2 removed, including investment costs (<10%), O&M costs (40%), electricity costs (<10%) for grinding and transport and distribution costs (~40% depending on distance)."

***Supplementary Table 1: Techno-economic parameters for bioenergy-based supply side technologies with carbon capture in REMIND**. Only when the captured CO_2 is geologically stored (and not used for synthetic fuels) will it be counted as CDR. REMIND tracks biomass flows in terms of energy content rather than tons of dry matter (tDM) and capture efficiencies in the model are given as Gigaton carbon per Zetajoule (GtC/ZJ) with respect to primary energy content. The carbon capture rates are calculated here for comparison using the assumptions of 18GJ/tDM on energy content per tonne dry matter and 0.49 tC/tDM on carbon content per tonne dry matter of biomass feedstock. *Capture rates of all technologies featuring carbon capture are additionally reduced by 1% to account for losses during CO_2 transportation, this table shows original capture rates before reduction.*

Bioenergy with carbon capture technology	Investment costs [US$2015/kW]	Fixed O&M costs as a fraction of investm. costs	Variable O&M costs [US$2015/kWa]	Energy conversion efficiency	Lifetime [years]	CO_2 capture GtC/ZJ* (rate)
**Electricity**: Biomass integrated gasification combined cycle power plant	3150	0.04	50.5	0.28	40	20<br>(73%)
**Hydrogen**	2040	0.08	10.6	0.55	35	22.5<br>(83%)
**Bioliquids:** Biodiesel production with Fischer-Tropsch	3600	0.06	10.6	0.41	35	12<br>(44%)
**Biogas**	1230	0.06	12.8	0.55	40	9.5<br>(35%)

Supplementary Table 2: Techno-economic parameters for Direct Air Capture in REMINDv3.2.0.

**DAC technoeconomic assumptions**	
Investment costs [US$2015/tC]	18000<br>(floor cost after learning: 4800)
Fixed O&M costs as a fraction of investm. costs	0.025
Energy demand (electr. for ventilation) [TWa/GtC]	5.28
Energy demand (heat for material recovery) [TWa/GtC]	21.12
Lifetime [years]	20

Supplementary Table 3: Techno-economic parameters for CO2 transport and storage in REMINDv3.2.0.

**CCS (geological storage of CO2) technoeconomic assumptions**	
Investment costs [US$2015/tC]	220
Fixed O&M costs as a fraction of investm. costs	0.06
Electricity demand [TWa/GtC]	0.005
Lifetime [years]	40
Total global storage potential GtCO2	3959
Maximum annual injection rate GtCO2/yr (0.5% of total storage potential)	19.9

***Supplementary Table 4: Assumptions on the regional potential of Enhanced Weathering.** Cropland area, maximum amount of ground stone on croplands, and maximum carbon removal potential for both warm and temperate climate zones for all REMIND model regions. A mapping of countries to the respective model region can be found in the Supplementary Material (page 3) from Strefler et al. 2021[4]. Supplementary Table 4 was directly taken from Strefler et al. 2021[4] Supplementary Information Table S4.*

Model region	Cropland area [10^6 km^2]		Maximum amount of ground stone on croplands [Gt]		Maximum carbon removal potential [Gt CO_2/yr]	
	**Warm**	**Temperate**	**Warm**	**Temperate**	**Warm**	**Temperate**
**CAZ**	0.00	0.14	0.03	2.06	0.00	0.04
**CHA**	0.40	0.65	5.96	9.74	0.33	0.17
**EUR**	0.00	0.69	0.06	10.35	0.00	0.18
**IND**	1.19	0.01	17.85	0.17	0.99	0.00
**JPN**	0.00	0.08	0.05	1.19	0.00	0.02
**LAM**	1.48	0.18	22.16	2.64	1.23	0.05
**MEA**	0.01	0.02	0.59	0.27	0.03	0.00
**NEU**	0.01	0.06	0.11	0.87	0.01	0.01
**OAS**	1.66	0.03	24.83	0.48	1.37	0.01
**REF**	0.00	0.73	0.00	10.98	0.00	0.19
**SSA**	0.36	0.04	4.92	0.56	0.27	0.01
**USA**	0.03	0.15	0.38	2.31	0.02	0.04
**Total**	5.13	2.77	76.91	41.60	4.25	0.71

Supplementary References

1. Luderer G, Bauer N, Baumstark L, et al. REMIND - REgional Model of INvestments and Development. Published online April 21, 2023. doi:10.5281/ZENODO.7852740

2. Baumstark L, Bauer N, Benke F, et al. REMIND2.1: Transformation and innovation dynamics of the energy-economic system within climate and sustainability limits. *Geosci Model Dev Discuss*. 2021;2021:1-50. doi:10.5194/gmd-2021-85

3. Beuttler C, Charles L, Wurzbacher J. The Role of Direct Air Capture in Mitigation of Anthropogenic Greenhouse Gas Emissions. *Front Clim*. 2019;1:10. doi:10.3389/fclim.2019.00010

4. Strefler J, Bauer N, Humpenöder F, Klein D, Popp A, Kriegler E. Carbon dioxide removal technologies are not born equal. *Environ Res Lett*. 2021;16(7):074021. doi:10.1088/1748-9326/ac0a11

5. Strefler J, Bauer N, Kriegler E, Popp A, Giannousakis A, Edenhofer O. Between Scylla and Charybdis: Delayed mitigation narrows the passage between large-scale CDR and high costs. *Environ Res Lett*. 2018;13(4):044015. doi:10.1088/1748-9326/aab2ba

6. Strefler J, Amann T, Bauer N, Kriegler E, Hartmann J. Potential and costs of carbon dioxide removal by enhanced weathering of rocks. *Environ Res Lett*. 2018;13(3):034010. doi:10.1088/1748-9326/aaa9c4
